# Nanocomposite Hydrogels Containing Few-Layer Graphene Sheets Prepared through Noncovalent Exfoliation Show Improved Mechanical Properties

**DOI:** 10.3390/nano12183129

**Published:** 2022-09-09

**Authors:** Ahmed El-Refaey, Yoshihiro Ito, Masuki Kawamoto

**Affiliations:** 1 RIKEN Center for Emergent Matter Science, 2-1 Hirosawa, Wako 351-0198, Saitama, Japan; 2 Graduate School of Science and Engineering, Saitama University, 255 Shimo-Okubo, Sakura-ku, Saitama 338-8570, Saitama, Japan; 3 Nano Medical Engineering Laboratory, RIKEN Cluster for Pioneering Research, 2-1 Hirosawa, Wako 351-0198, Saitama, Japan

**Keywords:** graphene, noncovalent functionalization, nanocomposite hydrogel

## Abstract

Hydrogels show great potential as soft materials for biomedical applications and flexible devices. However, conventional hydrogels exhibit poor mechanical strengths owing to the presence of water in their polymer networks. Therefore, improving the mechanical properties of hydrogels by controlling the chemical and physical structures that affect their macroscopic behaviors is a challenging issue. In this study, we developed a nanocomposite (NC) hydrogel that harbors exfoliated few-layer graphene sheets through noncovalent interactions. The bifunctional polymer PImQ, which contains both aromatic and cationic groups, was found to enable the direct exfoliation of graphite to few-layer graphene through π–π interactions in 2.7% yield. The poly(acrylamide)-based NC hydrogel containing the PImQ/graphene composite as a nanofiller shows a 3.4-fold increase in tensile stress compared with the hydrogel without the nanofiller. The introduction of the PImQ/graphene nanocomposite also increases the fracture stress of the NC hydrogel through cation–π and π–π interactions. The improved mechanical properties of the NC hydrogel result from the synergistic effects of the chemical crosslinking of the polymer network and the physical crosslinking of the polymer/graphene nanofiller.

## 1. Introduction

Hydrogels are typical soft materials consisting of three-dimensional polymer networks that exhibit excellent biocompatibility, permeability, and tissue-like properties [1,2,3]. Hydrogels have also been increasingly studied for application in flexible devices such as sensors [4], actuators [5], and soft robots [6]. Water-embedded hydrogel networks maintain solid-like integrity with flexibility. However, conventional hydrogels exhibit poor strain and strength behaviors.

Recently, nanocomposite (NC) hydrogels, which are reinforced with nano-sized fillers to improve their mechanical properties, have attracted extensive research attention [7,8]. Two-dimensional (2D) graphene nanosheets show promise as nanofillers for NC hydrogels. This nanomaterial can be obtained by exfoliating layered graphite structures. However, graphite is difficult to exfoliate owing to the substantial van der Waals interactions between the graphene sheets [9]. Although NC hydrogels containing graphene have been studied, most contain graphene oxide (GO) produced by chemical exfoliation [10,11,12].

The chemical exfoliation of graphite to obtain GO by oxidation is known as the Hummers’ method [13]. However, this covalent-functionalization approach produces structural defects comprising hydrophilic groups, where some of the hexagonal sp^2^ carbon atoms in the 2D lattice become sp^3^ hybridized. Such disturbances in the sp^2^-hybridized orbitals result in decreased electrical conductivity owing to disruption of the π-conjugated carbon structure.

In contrast, physical exfoliation of graphite through noncovalent interactions is a “soft” fabrication process [14]. Dispersants such as organic solvents [15], small molecules [16], surfactants [17], and polymers [18] are attached to the graphite surface by π–π and/or hydrophobic interactions, yielding suspensions of graphene sheets. Furthermore, physical exfoliation of graphite to few-layer graphene sheets has been achieved using polymer dispersants such as polyvinyl alcohol [19], bovine serum albumin [20], and polylactide [21]. Because minimal perturbation occurs in the electronic structure of the exfoliated graphene sheet, noncovalent exfoliation of graphite preserves the chemical structure of graphene [22]. However, hydrophobic graphene readily precipitates in water, yielding aggregated graphite. Therefore, both physical exfoliation and graphene-suspension stability are key challenges to manipulating 2D carbon nanosheets in water.

**Figure 1:**
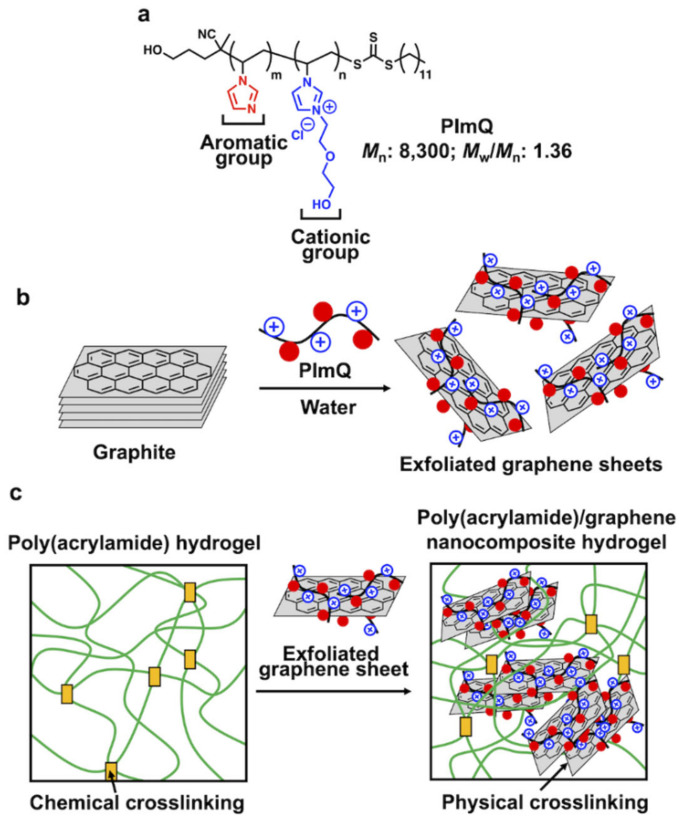
(**a**) Chemical structure of PImQ, which contains both aromatic and cationic groups. (**b**) Schematic of graphene sheets exfoliated from graphite in water through noncovalent interactions. (**c**) Poly(acrylamide)/graphene nanocomposite (NC) hydrogel with physical crosslinking between exfoliated graphene sheets.

In this study, we synthesized a polymer dispersant, PImQ, that contains imidazole moieties as aromatic groups and imidazolium moieties as cationic groups (Figure 1a). This bifunctional polymer allows both the physical exfoliation of graphite through noncovalent interactions and the stable suspension of graphene in water owing to electrostatic repulsion (Figure 1b). The aromatic groups in PImQ allow the exfoliation of graphite through π–π interactions with an exfoliation yield of 2.7%. Furthermore, the cationic groups in PImQ provide a positive zeta potential of +50 mV, inducing dispersion stability and preventing aggregation through electrostatic repulsion.

Here, PImQ/graphene is used as a nanofiller in NC hydrogels. The tensile stress of the NC hydrogel containing 3 wt% PImQ/graphene is 3.4 times that of the hydrogel without the PImQ/graphene nanofiller. Furthermore, the fracture stress of the hydrogel increases from 250 to 440 kPa upon the introduction of PImQ/graphene. This increase in mechanical strength is attributed to the physical crosslinking associated with the cation–π and π–π interactions of PImQ/graphene in the NC hydrogel (Figure 1c).

An important aspect of this study is that the exfoliated graphene sheets provided by PImQ change the macroscopic properties of the hydrogel depending on the assembled structure. The positively charged PImQ/graphene can be suspended in water owing to its electrostatic repulsion. In contrast, the cation–π and π–π interactions between the graphene sheets improve the mechanical properties of the NC hydrogel.

## 2. Materials and Methods

### 2.1. Materials

Unless otherwise noted, compounds and solvents were purchased from commercial suppliers and used without further purification. 1-Vinylimidazole and 2-(2-chloroethoxy)ethanol were purchased from Tokyo Chemical Industry Co., Ltd. (Tokyo, Japan). 2,2′-Azobis(2-methylpropionitrile) (AIBN), *N*,*N′*-methylene bis(acrylamide) (BIS), acrylamide (AAm), and anhydrous ethanol were purchased from FUJIFILM Wako Pure Chemical Co., Ltd. (Osaka, Japan). Acetic acid and diethyl ether were purchased from Junsei Chemical Co., Ltd. (Tokyo, Japan). 4-Cyano-4-[(dodecylsulfanylthiocarbonyl)sulfanyl]pentanol and 2-hydroxy-4′-(2-hydroxyethoxy)-2-methylpropiophenone were purchased from Sigma-Aldrich Co., Ltd. (St. Louis, MO, USA). Deuterated water was purchased from Cambridge Isotope Laboratories Inc. (Tewksbury, MA, USA). Graphite (≥99% carbon basis) was gifted from Nippon Graphite Industries Co., Ltd. (Shiga, Japan). Dialysis membranes were purchased from Repligen Co., Ltd. (Spectra/Por 3, MWCO: 3.5 kDa, Waltham, MA, USA). An elastic carbon-coated copper transmission electron microscopy (TEM) grid was purchased from Okenshoji Co., Ltd. (ELS-C10, Tokyo, Japan).

### 2.2. Preparation of PImQ/Graphene Suspensions

Graphite (5 mg) was added to deionized water containing PImQ (0.05 g L^−1^, 10 mL). The mixture was sonicated in an ice bath for 60 min using a tip-type ultrasonic homogenizer (Branson Sonifer 250, power output 20 W, Branson Ultrasonics, Danbury, CT, USA). The colloidal solution was centrifuged at 4000 rpm for 1 h using a microcentrifuge (Microfuge 16, Beckman Coulter, Brea, CA, USA). After centrifugation, homogeneous PImQ/graphene (0.1:1, (*w/w*)) was obtained from the supernatant. PImQ/graphene suspensions with different component ratios up to PImQ/graphene = 1:1 (*w/w*) were also prepared.

### 2.3. Preparation of NC Hydrogels Containing Exfoliated Graphene Sheets

Poly(acrylamide) (PAAm)-based NC hydrogels containing PImQ/graphene are denoted as G*x*H*y*, where *x* represents the weight percentage of PImQ/graphene (wt.%) and *y* represents the percentage molar ratio of the BIS crosslinker to AAm in NC hydrogels (%). For example, G0.2H0.05 means that the NC hydrogel is prepared with the weight percentage of PImQ/graphene of 0.2 wt.% and the molar ratio of BIS to AAm of 0.05%.

AAm (0.48 g, 17 mmol), BIS (0.05 mol% wrt. AAm, 2 mg), and 2-hydroxy-4′-(2-hydroxyethoxy)-2-methylpropiophenone (1 mol% wrt. AAm, 15 mg) were added to a PImQ/graphene suspension (0.5:1 *v/v*, 2 mL). We fabricated a cell from two glass substrates (cell area: 6 cm^2^, thickness: 1.2 mm). The cell was filled with the mixed solution by capillary action. Photopolymerization was performed by photoirradiation at 365 nm (light intensity: 4 mW cm^–2^) for 30 min. The resulting solid was removed from the glass cell and then washed with deionized water, yielding the graphene-doped hydrogel G0.2H0.05. NC hydrogels with different graphene ratios up to G3H0.05 were prepared using PImQ/graphene suspension = 5:1 (*v/v*).

### 2.4. Characterization

The synthesized compounds were structurally characterized by means of ^1^H and ^13^C NMR spectroscopy (AL 400, JEOL Ltd., Tokyo, Japan). *M*_w_ and *M*_n_ were measured by gel permeation chromatography (GPC; column: GF-510HQ, Showa Denko K. K., Tokyo, Japan) using 0.3 M NaCl_aq_ as the eluent, following calibration with pullulan standards. Absorption spectra of the exfoliated graphene were obtained using a UV–Vis spectrophotometer (V-750, Jasco Co. Ltd., Tokyo, Japan). The exfoliation yields for PImQ/graphene were estimated using thermogravimetric analysis (TGA; TG-DTA8122, heating rate: 5 °C min^−1^, RIGAKU Co., Tokyo, Japan). The exfoliated graphene was investigated using X-ray diffractometry (XRD; SmartLab, Rigaku, Tokyo, Japan), TEM (JEM-1230, accelerating voltage: 80 kV, JEOL Ltd., Tokyo, Japan), and Raman spectrometry (LabRAM, HORIBA Jobin Yvon GmbH, Bensheim, Germany). Zeta potentials were determined using a zeta potential analyzer (ELSZ-2PL, Otsuka Electronics Co. Ltd., Osaka, Japan). Photopolymerization for the preparation of hydrogels was carried out using a high-pressure mercury lamp (REX-250, Asahi Spectra Co. Ltd., Tokyo, Japan) equipped with a glass filter (HQBP365, Asahi Spectra Co. Ltd., Tokyo, Japan). The surface morphologies of the hydrogels were investigated using scanning electron microscopy (SEM; Quattro ESEM, accelerating voltage: 5 kV, Thermo Fisher Scientific, Waltham, MA, USA). Rheological properties were determined using a rheometer (AR-G2, TA instruments, New Castle, DE, USA) with a 25 mm diameter parallel plate attached to a transducer. Mechanical tensile-stress analyses were performed using a testing machine (model 3345, Instron, Norwood, MA, USA). Samples were evaluated for each graphene loading amount. Tensile analyses were conducted at 25 °C with different strain rates to evaluate stretchability according to the ASTM 882-09 test method using dumbbell configured specimens according to JIS K-6251-7 (width: 2 mm; length: 12 mm; thickness: 1.2 mm). Experimental values for strain-at-break and stress-at-break were obtained by uniaxial tensile testing at a strain rate of 100 mm min^−1^.

## 3. Results and Discussion

### 3.1. Noncovalent Exfoliation of Graphite to Graphene Using PImQ

#### 3.1.1. Aqueous Suspension of PImQ/Graphene

PImQ was synthesized using reversible addition−fragmentation chain-transfer (RAFT) polymerization (see Supplementary Materials). The RAFT polymerization of 1-vinyl imidazole, 4-cyano-4-[(dodecyl-sulfanylthiocarbonyl) sulfanyl] pentanol, and AIBN in CH_3_COOH produced poly(1-vinylimidazole) (PIm) (Figure S1). After purification of PIm by dialysis, 2-(2-chloroethoxy) ethanol was dissolved in EtOH and reacted at 70 °C for 24 h to obtain PImQ. ^1^H NMR methylene peaks at 3.48–3.78 ppm in D_2_O confirmed that quaternization had occurred (Figure S2).

**Figure 2:**
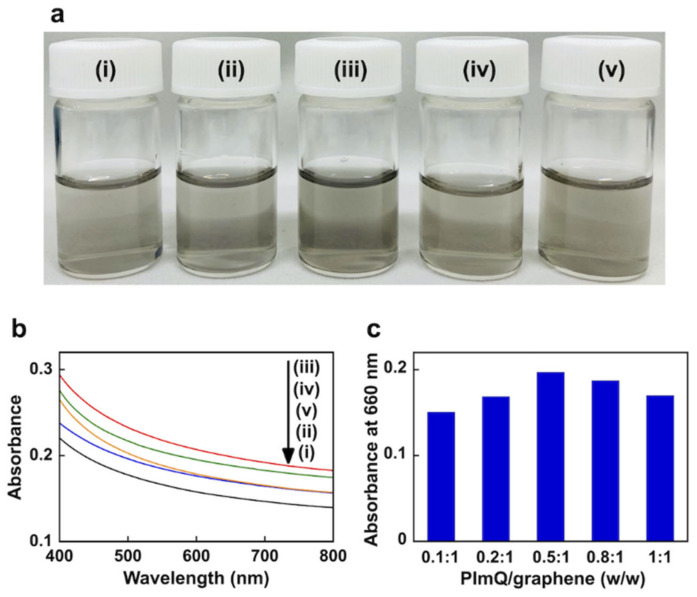
(**a**) Photographs of aqueous exfoliated graphene suspensions with different concentrations of PImQ. (**b**) Absorption spectra of PImQ/graphene suspensions in water. (**c**) Comparison of absorbance at 660 nm for each ratio of PImQ/graphene. PImQ/graphene = (i) 0.1:1 (*w/w*), (ii) 0.2:1 (*w/w*), (iii) 0.5:1 (*w/w*), (iv) 0.8:1 (*w/w*), (v) 1:1 (*w/w*).

Figure 2a shows images of graphene suspensions in water exfoliated with various concentrations of PImQ. As the concentration of PImQ increases, the suspensions become darker. The absorption spectra show an increase in absorbance up to PImQ/graphene = 0.5:1 (*w/w*); however, absorbance decreases at higher concentrations of PImQ (Figure 2b). These results indicate that PImQ acts as a dispersant for exfoliated graphene and that its dispersing ability is saturated at high concentrations.

The extinction coefficient (*α*_G_) of exfoliated graphene was measured at 660 nm, which is commonly used for graphene analysis [23,24,25]. In addition, PImQ has no light absorption at 660 nm, facilitating the determination of *α*_G_. Figure 2c shows absorbance at 660 nm (*A*_660_) for PImQ/graphene composites with different component ratios. According to the Beer–Lambert law, *A*_660_ = *α*_G_C_G_*l*, the concentration of exfoliated graphene (*C*_G_) is proportional to the absorbance per pass length *l* (*A*_660_ *l*^−1^). The *α*_G_ value was calculated to be 1216 mL mg^−1^ m^−1^ using the following parameters: *C*_G_ = 0.0162 mg mL^−1^ and *A*_660_ *l*^−1^ = 19.7 m^−1^. This value is similar to the *α*_G_ value of 1390 mL mg^−1^ m^−1^ reported in a previous study [17]. *A*_660_ for the PImQ/graphene (0.5:1 (*w/w*)) suspension remains unchanged during one month of storage at 25 °C, indicating that this sample exhibits stable suspension ability (Figure S5).

Furthermore, we determined the exfoliation yield of graphite to clarify the exfoliation ability of PImQ using thermogravimetric analysis (TGA). The exfoliation yield is 2.7% for PImQ/graphene (0.5:1 (*w/w*)) (Figure S6). This value is higher than those for conventional dispersants such as surfactants (0.36%) [17], peptides (1.2%) [26], and liposomes (2.5%) [27], demonstrating that PImQ has good graphite exfoliation ability.

Zeta potential (*ζ*) analysis was used to investigate the dispersion behavior of the PImQ/graphene suspension. We found that the suspension exhibits dispersion stability in water. The *ζ* value of the PImQ solution is +38 mV owing to the cationic groups of the imidazolium units in the polymer side chains (Figure S7a). In contrast, a PImQ/SWCNT suspension presents the high *ζ* of +50 mV in water (Figure S7b). These results suggest that the noncovalent interactions of PImQ with the graphene surface provide a positive interface potential, resulting in the aqueous dispersion of graphene sheets by electrostatic repulsion.

#### 3.1.2. Exfoliation Behavior of the PImQ/Graphene Sheets

**Figure 3:**
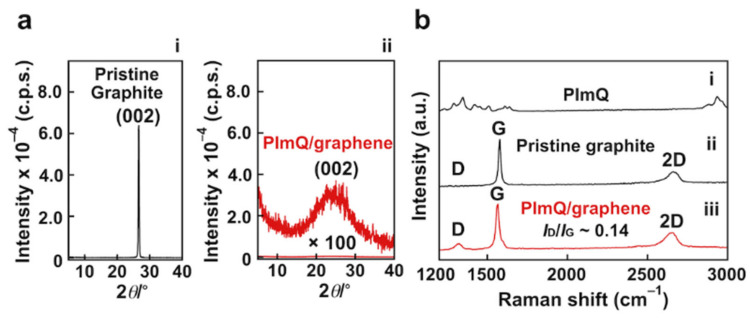
(**a**) XRD patterns of (i) pristine graphite and (ii) PImQ/graphene. (**b**) Raman spectra of (i) PImQ, (ii) pristine graphite, and (iii) PImQ/graphene. Excitation wavelength for Raman spectra: 633 nm.

To evaluate exfoliation behavior, we used XRD analysis (Figure 3a). Pristine graphite shows a diffraction peak at 2*θ* = 26.5° corresponding to the (002) reflection owing to the 3.34 Å separation between graphene layers (Figure 3a(i)). The intensity of the peak at 26.5° for PImQ/graphene is decreased by 99% compared with that for graphite. This result indicates that graphite is exfoliated by charge repulsion in the presence of cationic PImQ, yielding the random structure of the graphene sheet (Figure 3a(ii)).

Raman spectroscopy was used to assess the quality of the graphene sheets provided by noncovalent exfoliation (Figure 3b). The characteristic bands for imidazole and imidazolium ring vibrations in PImQ appear at 1300–1600 cm^−1^ [28]. The C–H stretching modes present a complex pattern formed by overlapping bands in the high-frequency range 2800–2950 cm^−1^ (Figure 3b(i)). The characteristic bands of graphene are observed, i.e., the defect-induced (D) band (1323 cm^−1^); the (G) band (1567 cm^−1^), which corresponds to the vibration of sp^2^ carbons in the graphitic layers; and the 2D band, which is derived from two-photon modes of graphene sheets (2652 cm^−1^) (Figure 3b(ii)). The D/G band intensity ratio (*I*_D_/*I*_G_) is an important parameter that indicates the degree of structural defect in graphene. *I*_D_/*I*_G_ for the exfoliated graphene is 0.14, which is lower than those for graphene chemically exfoliated using sodium hydride (*I*_D_/*I*_G_: 1.08) [29] and hydrazine (*I*_D_/*I*_G_: 1.44) [30] (Figure 3b(iii)). These results indicate that the graphene sheets produced by PImQ through noncovalent exfoliation are of high-quality with few structural defects.

**Figure 4:**
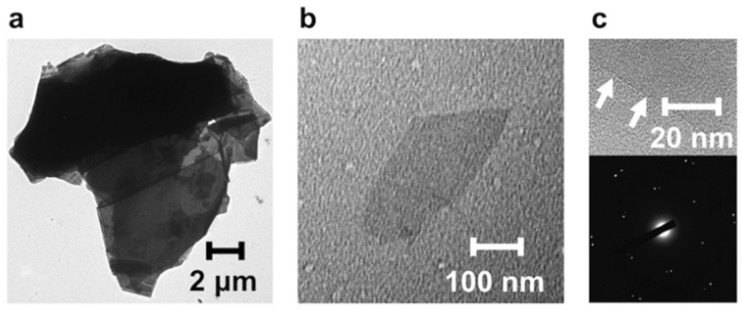
(**a**) TEM image of pristine graphite. (**b**) TEM image of a graphene sheet exfoliated using PImQ. (**c**) HR-TEM image and electron diffraction pattern for the edge of an exfoliated graphene sheet. Arrows indicate the edges of exfoliated graphene sheets.

TEM was used to observe an exfoliated graphene sheet directly. It is important to understand the exfoliated graphene that remains dispersed after centrifugation. First, the initial state of the pristine graphite was examined (Figure 4a). TEM observation revealed that the graphite flakes form aggregates with a lateral size of ~12 μm. In contrast, the exfoliated PImQ/graphene after centrifugation contains sheets 200–400 nm in size (Figure 4b). We found that ultrasonication fragments pristine graphite and centrifugation removes the large aggregates. This sonication-induced fragmentation of graphene is similar to that of carbon nanotubes [31]. The high-resolution TEM (HR-TEM) image shows bright edges and clear dark lines, characteristic of few-layer graphene sheets (Figure 4c). Furthermore, the electron diffraction pattern reveals typical six-fold symmetry attributed to graphite/graphene. These peaks could be labeled with the Miller–Bravais (*hkil*) index through (1–210)–(0–110)–(–1010)–(–2110) diffractions [32]. We also observed two sets of hexagonal spots in the diffraction pattern. This Moiré pattern indicates the presence of few-layer graphene sheets in which multiple graphene layers overlap at different angles [33]. These results suggest that noncovalent exfoliation using PImQ produces few-layer graphene sheets with few edge defects. It should also be noted that the *α*_G_ value derived from the Beer–Lambert law was obtained from few-layer graphene sheets.

### 3.2. Mechanical Properties of NC Hydrogels Containing Exfoliated Graphene Sheets

#### 3.2.1. Rheological and Mechanical Behavior of PAAm Hydrogels

**Figure 5:**
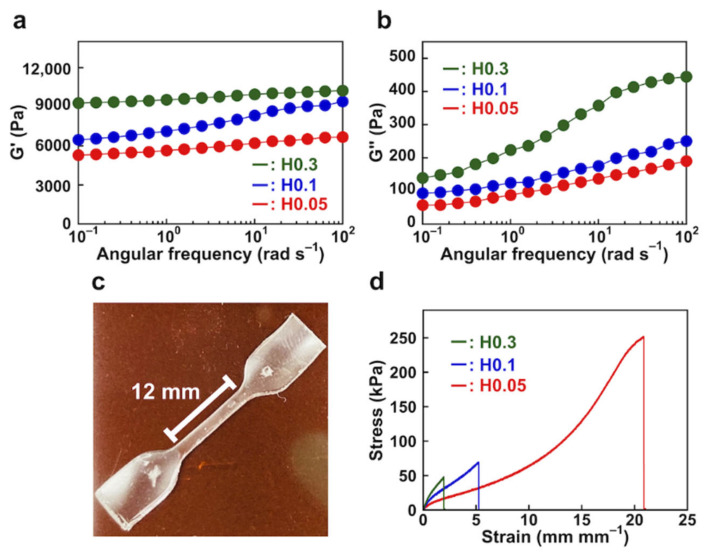
(**a**) Storage modules (G′) and (**b**) loss modules (G″) for PAAm gels recorded at 25 °C. (**c**) Dumbbell-shaped specimen used for stress–strain measurements. (**d**) Stress–strain curves for PAAm gels with a strain rate of 100 mm min^−1^ at 25 °C.

First, we investigated the rheological properties of PAAm gels without PImQ/graphene. The storage modulus (G′) increases with increasing concentration of the crosslinker in the frequency dispersion curve (Figure 5a). This result indicates that a high concentration of crosslinker in the hydrogel imparts an ability to store the energy generated by internal strain. In contrast, the loss modulus (G″) curves show no signs of relaxation and only shallow minima, suggesting that the PAAm gels contain chemical networks (Figure 5b).

To confirm these properties, we performed mechanical tensile stress analysis of the PAAm gels. A dumbbell-shaped hydrogel (width: 2 mm; length: 12 mm; thickness: 1.2 mm) was clamped in a tensile testing machine and stressed at a rate of 100 mm min^−1^ (Figure 5c). The H0.05 hydrogel with a crosslinker concentration of 0.05% exhibits remarkable elasticity, with a tensile strength of 250 kPa and an elongation-at-break of 2100% (Figure 5d). The elasticity for the H0.3 hydrogel with the crosslinker concentration of 0.3% is significantly lower and its elongation-at-break is 207%. Thus, a higher concentration of crosslinker produces tight chemical crosslinking, resulting in a more rigid hydrogel.

#### 3.2.2. Surface Morphologies of NC Hydrogels Containing PImQ/Graphene Nanofillers

**Figure 6:**
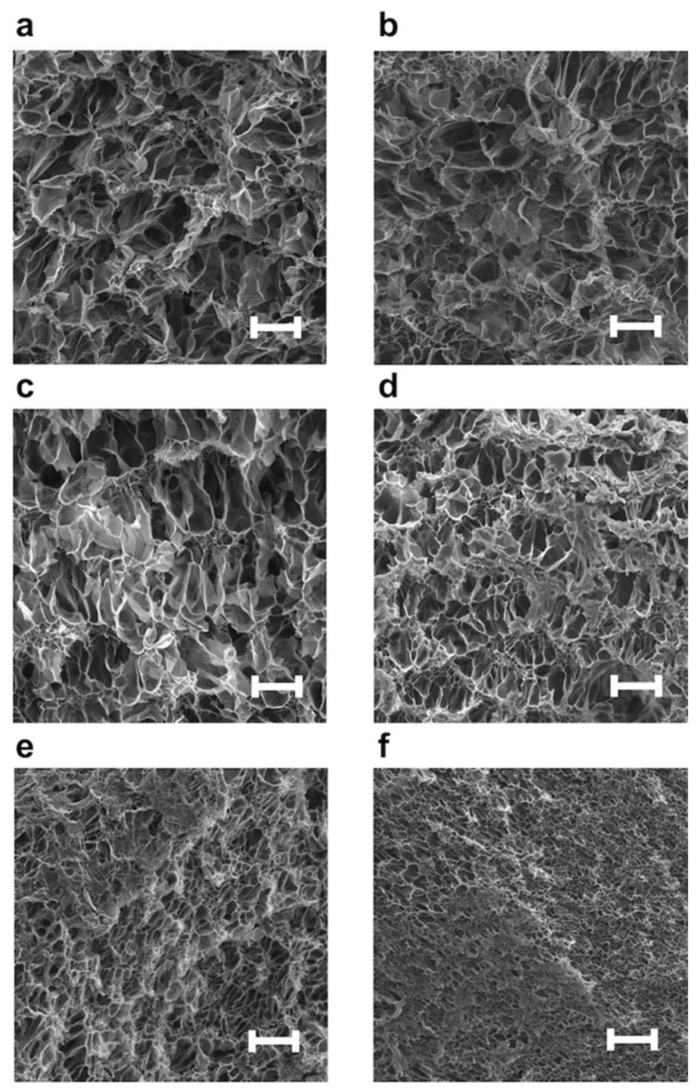
SEM images of PAAm and NC hydrogels containing PImQ/graphene. (**a**) H0.05, (**b**) G0.2H0.05, (**c**) G0.5H0.05, (**d**) G1H0.05, (**e**) G2H0.05, (**f**) G3H0.05. Scale bars are 20 μm.

The SEM images show that the microstructures of freeze-dried NC hydrogels are highly dependent on the concentration of PImQ/graphene (Figure 6). To confirm the effect of adding PImQ/graphene, H0.05, which exhibits the highest elongation-at-break (Figure 5d), was used as the standard hydrogel. The microstructures of the freeze-dried hydrogels feature pores created by swollen ice crystals. In H0.05 without PImQ/graphene, the presence of chemically crosslinked PAAm chains results in interconnected mesh-like microstructures in the swollen hydrogel (Figure 6a). SEM images of G0.2H0.05 and G0.5H0.05 with small amounts of PImQ/graphene as nanofiller show microstructures similar to that of H0.05 (Figure 6b,c). In contrast, the number of interconnected pores in the NC hydrogel increases and the pore size decreases when the concentration of PImQ/graphene increases beyond 1 wt% (Figure 6d–f). These results suggested that PImQ/graphene in hydrogels forms aggregated structures through cation–π and π–π interactions, which provide physical crosslinking in the polymer networks. This physical crosslinking changes the mechanical properties of the NC hydrogels.

#### 3.2.3. Mechanical Properties of NC Hydrogels

**Figure 7:**
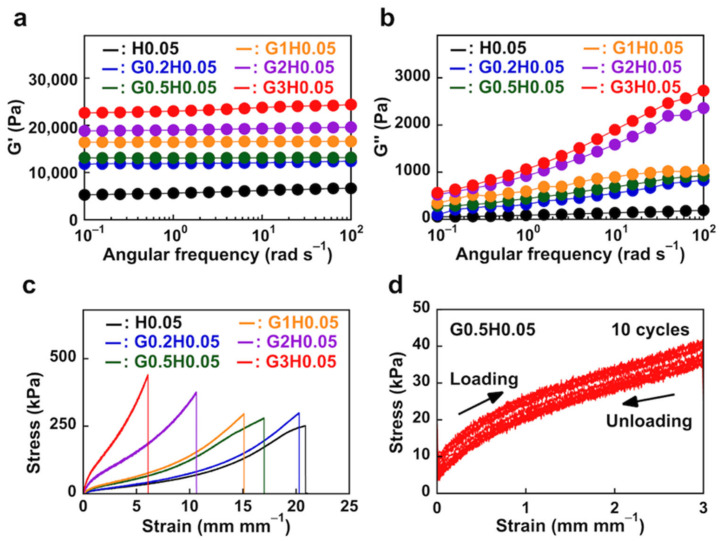
(**a**) Storage moduli (G′) and (**b**) loss moduli (G″) of NC hydrogels at 25 °C. (**c**) Stress–strain curves for NC hydrogels with a strain rate of 100 mm min^−1^ at 25 °C. (**d**) Cyclic tensile loading−unloading curves for G0.5H0.05 at 300% tensile strain.

We expected that the physical crosslinking of the PImQ/graphene would alter the viscoelasticity of the NC hydrogel. Rheological tests showed that the NC hydrogel with PImQ/graphene exhibits enhanced viscoelasticity, confirming the crosslinked structure of the network. The G′ value for G3H0.05 is ~3.6 times higher than that for H0.05, indicating that the cation–π and π–π interactions in PImQ/graphene promote physical interactions within the polymer network (Figure 7a). The change in the value of G″ with respect to the amount of PImQ/graphene introduced is even more pronounced. In particular, the G″ value for G3H0.05, which has a high concentration of PImQ/graphene, is 14 times higher than that for H0.05, providing good elastic properties (Figure 7b). Furthermore, large changes in G″ are observed for G1H0.05 and G2H0.05. This result is presumably similar to the multivalent effect observed for NC hydrogel containing inorganic clay sheets [34]. As the PImQ/graphene concentration increases, the resulting NC hydrogel exhibits a multivalent effect owing to physical interactions, leading to an increase in dynamic viscoelasticity.

The tensile stresses for the G*x*H0.05 NC hydrogels are higher than that for the H0.05 hydrogel owing to the physical interactions formed between the crosslinked polymer chains (Figure 7c). For G*x*H0.05 hydrogels with different concentrations of PImQ/graphene, the Young’s modulus increases with increasing graphene content at 300% tensile strain (Figure S8). This suggests that PImQ/graphene acts as a physical crosslinker that dissipates strain energy in the polymer network and effectively toughens the G*x*H0.05 NC hydrogels. We also found that the mechanical strength of NC hydrogels with PImQ/graphene is better than that of NC hydrogels with GO. The stress of the G3H0.05 NC hydrogel is 440 kPa at 610% strain, while that of a previously reported NC hydrogel containing GO was approximately 180 kPa at 60% strain [11]. This indicates that PImQ/graphene as a physical crosslinker significantly improves the mechanical strength of the NC hydrogel. Furthermore, increasing the PImQ/graphene content decreases the fracture strain of the hydrogel. When the physical crosslinking of the PImQ/graphene between PAAm chains through cation–π and π–π interactions is enhanced, hydrogel extensibility decreases with increasing crosslinking density. Thus, the mechanical strength of the hydrogels can be controlled by altering the concentration of PImQ/graphene. We speculate that the mechanical behavior of the NC hydrogels is dictated by the synergistic effect of chemical crosslinking between the PAAm chains and the physical crosslinking within PImQ/graphene. Thus, controlling the amount of PImQ/graphene changes how the NC hydrogel dissipates energy during deformation.

Figure 7d shows the results of cyclic loading–unloading testing of a G0.5H0.05 NC hydrogel at 300% tensile strain. During the loading–unloading test, no rest period was applied over 10 cycles. G0.5H0.05 exhibits a distinct hysteresis in the loading cycle. After 10 cycles, the change in tensile stress is approximately 12%, indicating that the NC hydrogel exhibits the mechanical stability with recovery strain of 300% without fracture upon deformation. These results indicate that the chemical and physical crosslinkers in the NC hydrogel impart restoration ability against structural deformation. Conventional hydrogels break at pressures of several kilopascals because of the low density and low friction of polymer chains. In contrast, the NC hydrogels containing the PImQ/graphene nanofiller exhibit both flexibility and resilience owing to physical crosslinking through cation–π and π–π interactions. This presents the possibility of developing highly functional soft materials by controlling the molecular structures of chemical crosslinkers and the aggregation of physically crosslinked graphene nanosheets.

## 4. Conclusions

We prepared NC hydrogels containing few-layer graphene sheets exfoliated from graphite by noncovalent interactions. PImQ/graphene acts as a physical crosslinker in the hydrogel based on cation–π and π–π interactions. These physical interactions are affected by the concentration of PImQ/graphene and significantly change the tensile strength of the NC hydrogels. We also found that the mechanical strength of the NC hydrogels can be controlled by altering the concentration of PImQ/graphene. This mechanical behavior results from the synergistic effects of the chemical and physical crosslinking in the hydrogel.

## Data Availability

The data presented in this study are available on request from the corresponding authors.

## Supplementary Materials

The following supporting information can be downloaded at: https://www.mdpi.com/article/10.3390/nano12183129/s1, Scheme S1: Synthesis of poly(*N*-vinylimidazole) (PIm); Scheme S2: Synthesis of quaternized PIm (PImQ); Figure S1: ^1^H NMR spectrum of PIm in D_2_O; Figure S2: ^1^H NMR spectrum of PImQ in D_2_O; Figure S3: ^13^C NMR spectrum of PIm in D_2_O; Figure S4: ^13^C NMR spectrum of PImQ in D_2_O; Figure S5: A_660_ values of a PImQ/graphene (0.5:1 (*w/w*)) suspension plotted against storage time; Figure S6: TGA profiles of (a) graphite and PImQ and (b) PImQ/graphene (0.5:1 (*w/w*)) obtained at a heating rate of 5 °C min^−1^ under nitrogen; Figure S7: Zeta potentials of (a) PImQ and (b) PImQ/graphene (0.5:1 (*w/w*)) in water; Figure S8: Effects of PImQ/graphene concentration on Young’s moduli for nanocomposite hydrogels (tensile strain of 300%, at 25 °C).
