# Supplementary material for: Nanocomposite Hydrogels Containing Few-Layer Graphene Sheets Prepared through Noncovalent Exfoliation Show Improved Mechanical Properties
Source: Nanomaterials (Basel). 2022 Sep 9;12(18):3129. doi: 10.3390/nano12183129 (PMC9505845; doi:10.3390/nano12183129)
Supplement: Supplementary file 1 [file nanomaterials-12-03129-s001.zip › nanomaterials-1874058-supplementary.pdf]

nanomaterials

MDPI

Supplementary Materials:

Nanocomposite Hydrogels Containing Few-Layer Graphene Sheets Prepared through Noncovalent Exfoliation Show Improved Mechanical Properties

1. Synthesis of PImQ

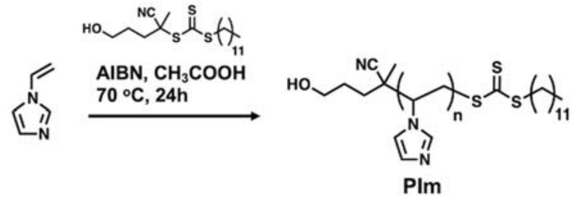

Scheme S1. Synthesis of poly(*N*-vinylimidazole) (PIm).

1-vinyl imidazole (2.0 g, 21 mmol) was added to 4-cyano-4-[(dodecyl-sulfanylthiocarbonyl) sulfanyl] pentanol (83 mg, 0.21 mmol) and 2,2′-azobis(2-methylpropionitrile) (17 mg, 0.1 mmol) in acetic acid (8 mL). The reaction mixture was degassed by three freeze–evacuate–thaw cycles under argon. The solution was stirred at 70 °C for 24 h under argon. After cooling to 25 °C, the resulting solution was poured into diethyl ether (200 mL). The precipitate was collected by filtration under reduced pressure. The solid residue was purified by dialysis against water for 72 h. The product was obtained after freeze-drying, yielding PIm (1.1 g, conversion: 57%) as a white powder. ^{1}H-NMR (400 MHz, D_2O): δ = 0.74-1.61 ($\mathrm{HOCH_2C}\mathit{H}_2\mathrm{C}\mathit{H}_2\mathrm{C(C}\mathit{H}_3\mathrm{)(CN)CH}$-imidazole-$\mathrm{CH_2SCSSCH_2CH_2C_{10}}\mathit{H}_{21}$)), 1.88-2.12 ($\mathrm{HOCH_2CH_2CH_2C(CH_3)(CN)CH}$-imidazole-$\mathrm{C}\mathit{H}_2\mathrm{SCSSCH_2C}\mathit{H}_2\mathrm{C_{10}H_{21}}$)), 2.53-3.76 ($\mathrm{HOC}\mathit{H}_2\mathrm{CH_2CH_2C(CH_3)(CN)C}\mathit{H}$-imidazole-$\mathrm{CH_2SCSSC}\mathit{H}_2\mathrm{CH_2C_{10}H_{21}}$), 6.63-7.21 (aromatic ring); ^{13}C-NMR (100 MHz, D_2O): δ = 14.0, 23.3, 29.7, 40.0, 40.5, 50.8, 51.2, 52.2, 52.4, 54.0, 54.1, 116.9, 117.1, 117.2, 117.4, 117.7, 117.9, 128.0, 128.8, 129.3, 136.1, 136.6, 136.8, 137.0, 137.2.

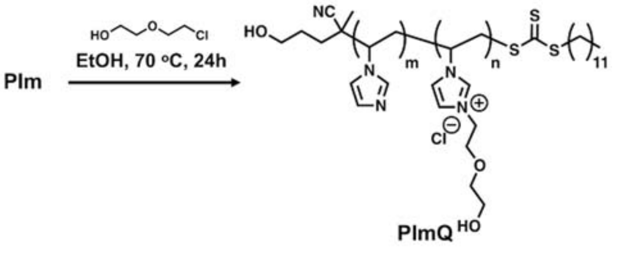

Scheme S2. Synthesis of quaternized PIm (PImQ).

PIm (0.29 g) and 2-(2-chloroethoxy) ethanol (12 mg, 0.09 mmol) were dissolved in anhydrous ethanol (6.5 mL). The reaction mixture was degassed by three freeze–evacuate–thaw cycles under argon. The solution was stirred at 70 °C for 24 h under argon. After cooling to 25 °C, the resulting solution was poured into diethyl ether (150 mL). The precipitate was washed with diethyl ether and collected by filtration under reduced pressure. The solid residue was dried in a vacuum oven at 50 °C for 24 h to yield PImQ (0.36 g). ^{1}H-NMR (400 MHz, D_2O):
δ = 0.82-1.64 ($\mathrm{HOCH_2C}\mathit{H}_2\mathrm{C}\mathit{H}_2\mathrm{C(C}\mathit{H}_3\mathrm{)(CN)CH}$-imidazole-$\mathrm{CH_2CH}$-imidazolium($\mathrm{CH_2CH_2OCH_2CH_2OH}$)-$\mathrm{CH_2SCSSCH_2CH_2C_{10}}\mathit{H}_{21}$)), 2.03-2.37 ($\mathrm{HOCH_2CH_2CH_2C(CH_3)(CN)CH}$-imidazole-$\mathrm{C}\mathit{H}_2\mathrm{CH}$-i-idaz-lium($\mathrm{CH_2CH_2OCH_2CH_2OH}$)-$\mathrm{C}\mathit{H}_2\mathrm{SCSSCH_2C}\mathit{H}_2\mathrm{C_{10}H_{21}}$)), 2.58-4.32 ($\mathrm{HOC}\mathit{H}_2\mathrm{CH_2CH_2C(CH_3)(CN)C}\mathit{H}$-imidazole-$\mathrm{CH_2C}\mathit{H}$-

imidazolium($CH_2CH_2OCH_2CH_2OH$)-$CH_2SCSSCH_2CH_2C_{10}H_{21}$)), 6.56-8.71 (aromatic ring); ^{13}C-NMR (100 MHz, D_2O): δ = 40.1, 48.9, 49.3, 49.6, 52.6, 53.0, 60.3, 67.7, 71.7, 117.0, 117.5, 120.0, 123.9, 127.9, 129.0, 129.7, 135.1, 136.5, 136.8; number-average molecular weight (M_n): 8300, weight-average molecular weight (M_w)/M_n: 1.36.

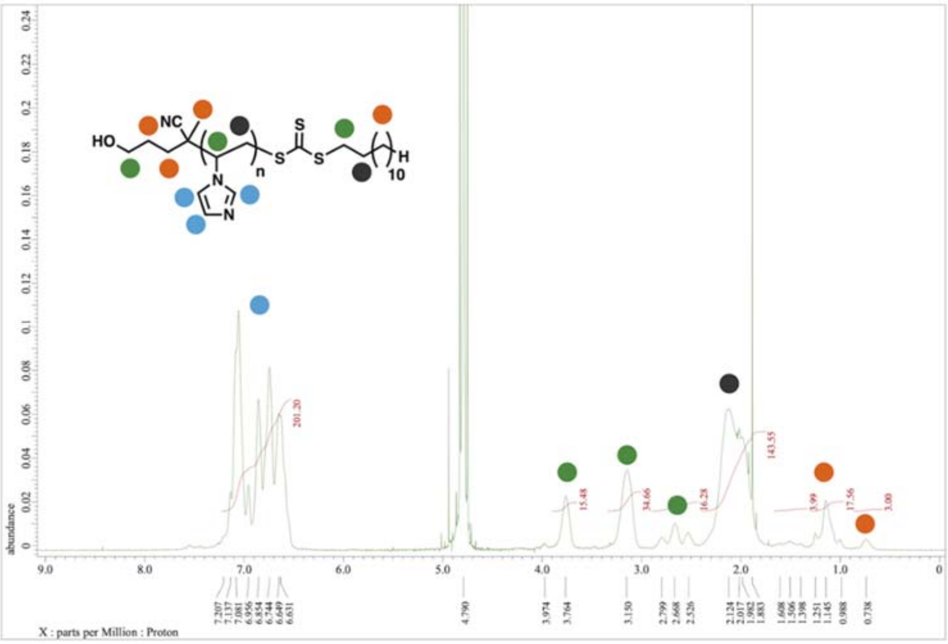

Figure S1. ^{1}H NMR spectrum of PIm in D_2O.

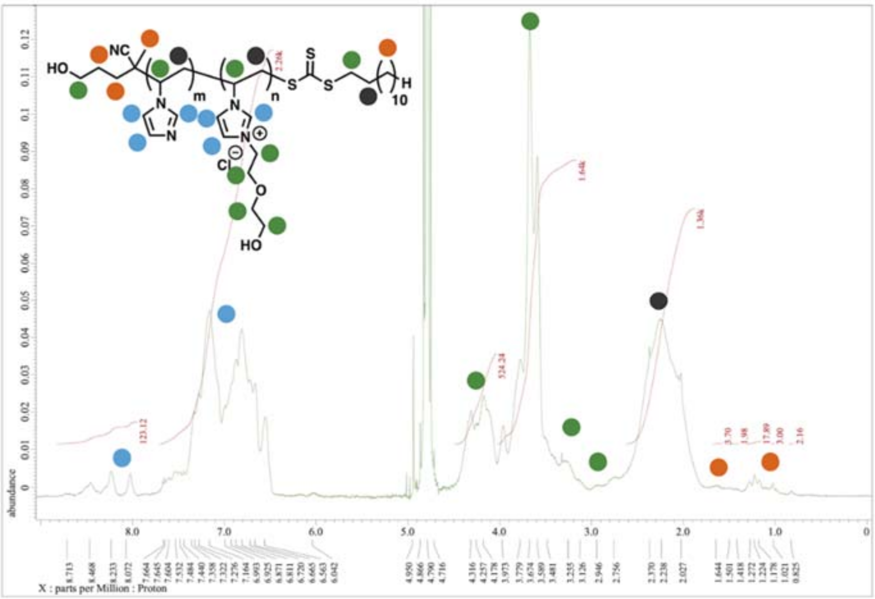

Figure S2. ^{1}H NMR spectrum of PImQ in D_2O.

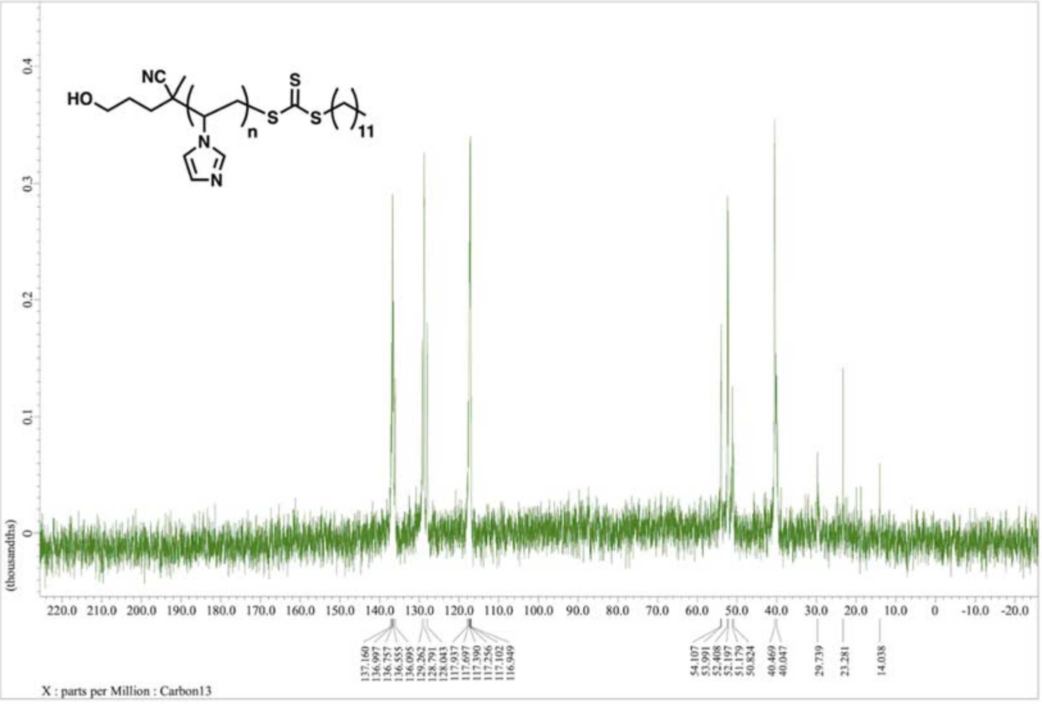

Figure S3. ^{13}C NMR spectrum of PIm in D_2O.

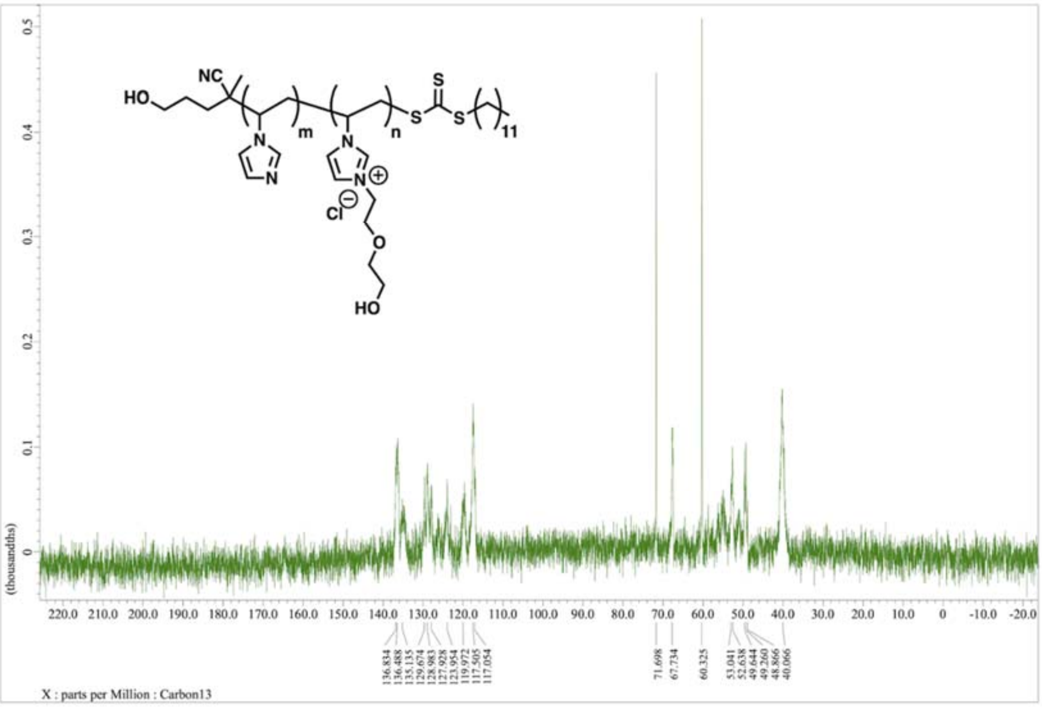

Figure S4. ^{13}C NMR spectrum of PImQ in D_2O.

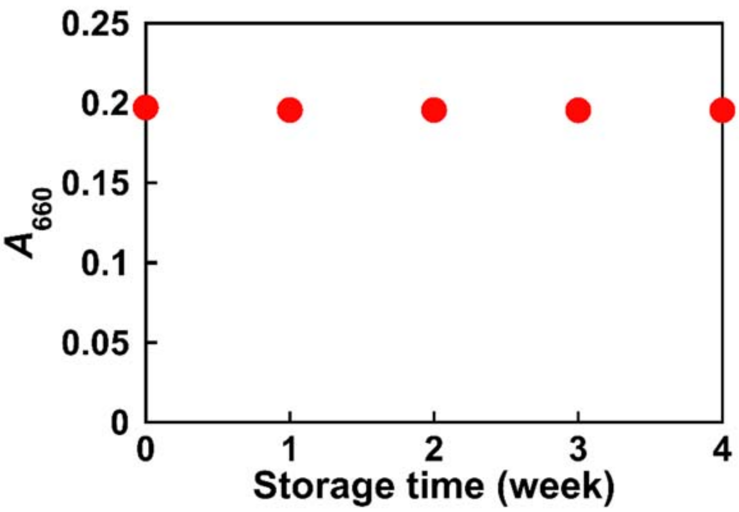

Figure S5. A_{660} values of a PImQ/graphene (0.5:1 (*w*/*w*)) suspension plotted against storage time.

The exfoliation yield for PImQ/graphene was determined using thermogravimetric analysis (TGA). Figure S6a shows the TGA profiles of graphite and PImQ recorded at a heating rate of 5 °C min^{-1} under nitrogen. Graphite exhibits no weight loss between 25 and 1000 °C. In contrast, PImQ exhibits thermal decomposition with a weight loss of 98.9% upon heating at 1000 °C.

We investigated thermal behavior of PImQ/graphene (0.5:1 (*w*/*w*)) using the same experimental conditions (Figure S6b).

Initial amount of PImQ/graphene at 25 °C: 2.58 mg

Residual amount of PImQ/graphene after heating at 1000 °C: 0.138 mg

Residual graphene was calculated to be 0.138 × 0.989 = 0.136 mg.

Since the initial amount of graphite is 5.0 mg before exfoliation, the exfoliation yield is (0.136/5.0) × 100 = 2.7%.

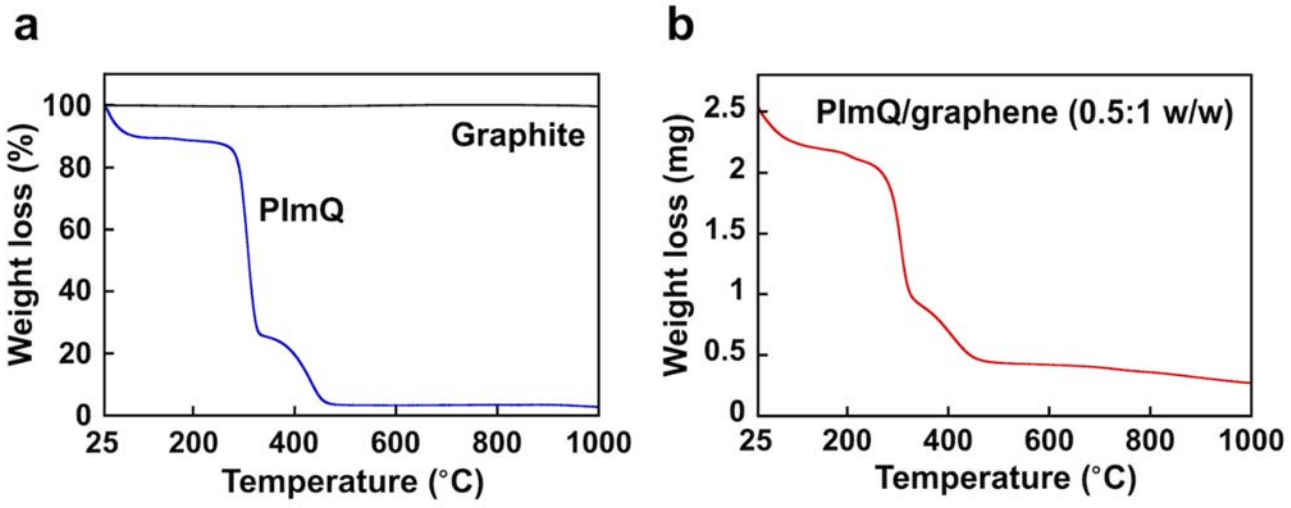

Figure S6. TGA profiles of (**a**) graphite and PImQ and (**b**) PImQ/graphene (0.5:1 (*w*/*w*)) obtained at a heating rate of 5 °C min^{-1} under nitrogen.

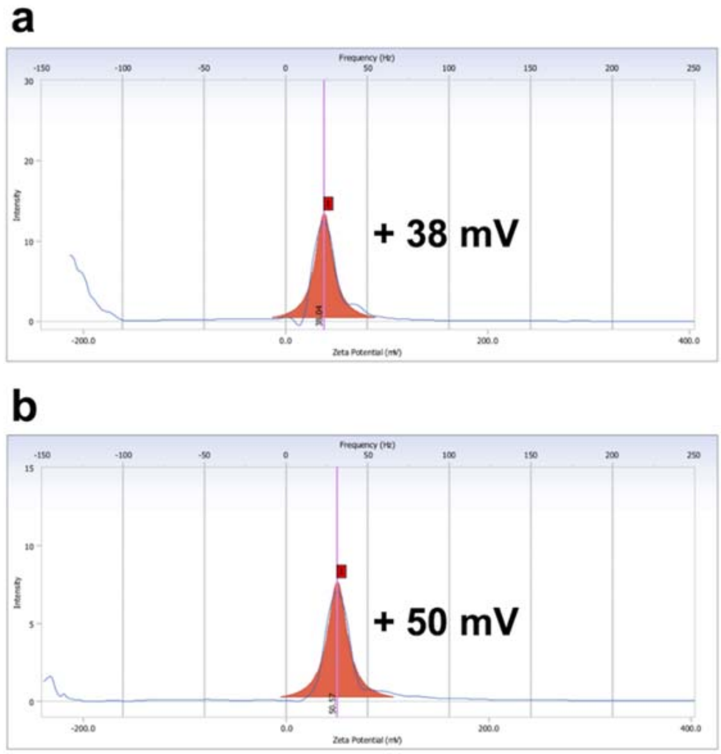

Figure S7. Zeta potentials of (a) PImQ and (b) PImQ/graphene (0.5:1 (w/w)) in water.

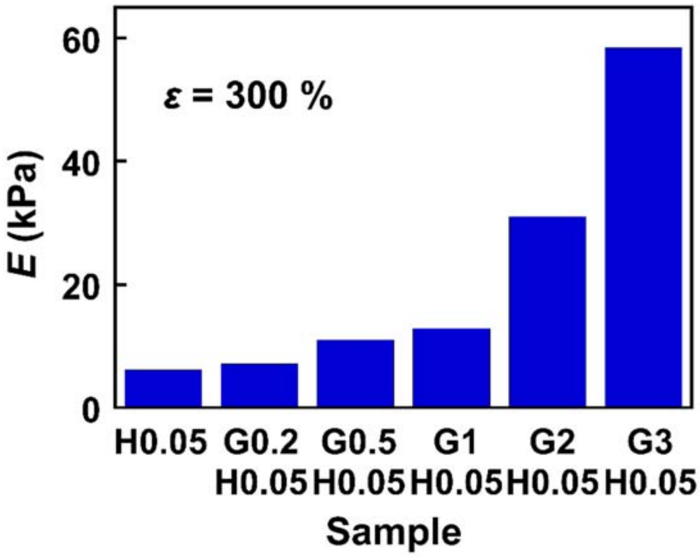

Figure S8. Effects of PImQ/graphene concentration on Young's moduli for nanocomposite hydrogels (tensile strain of 300 % at 25 °C).
